# Impact of leptin or melatonin on Sema4D overexpression-related bone metabolism

**DOI:** 10.1186/s13018-023-03740-6

**Published:** 2023-04-08

**Authors:** Zhenen Lin, Shengren Xiong, Yu Lin, Zhaohui Li, Dan Xie, Xuchao Lin, Xuesheng Chen, Xueyi Lin

**Affiliations:** Department of Orthopaedics, Fuzhou Second Hospital, 47th Shangteng Road of Cangshan District, Fuzhou, 350007 China

**Keywords:** Sema4d, Leptin, Melatonin, Osteoporosis, Bone metabolism

## Abstract

**Purpose:**

The current study aims to investigate the regulatory impact of leptin or melatonin on bone metabolism as well as the underlying mechanism in conjunction with Sema4D (monoclonal antibody to semaphorin 4D).

**Methods:**

Rats were used to create the osteoporosis model utilizing the OVX (OVariectomize) technique. Rat tibial specimens from each side were collected for three-dimensional reconstruction and Micro-CT scanning examination. The Hematoxylin-osinstaining (HE) staining technique was used to determine the pathological condition of bone tissues. The ELISA (Enzyme-Linked Immunosorbent Assay) assay was used to measure the amount of estradiol present in the serum. In the current study, there were six groups: control, OVX, OVX + NL (no load group), OVX + Sema4D, OVX + Sema4D + leptin, and OVX + Sema4D + MT (melatonin). Rats were given injections of the Sema4D or leptin overexpressing vectors via the tail vein in accordance with the aforementioned classification. By using a high-resolution micro-CT technology, 3D bone structure was discovered. The activity of tartrate-resistant acid phosphatase-5b (TRAP-5b) and bone-derived alkaline phosphatase (BALP) in serum was assessed using an ELISA. The number of osteoclasts in the metaphysis of the upper tibia was determined using TRAP (tartrate-resistant acid phosphatase) staining. Immunohistochemistry was used to find leptin and bone morphogenetic protein-2 (BMP-2) expressions in bone tissue.

**Results:**

The BV/TV (Bone volume/Tissue volume), Tb.N (Trabecular number), BMD (Bone Mineral Density), and BMC (Bone Mineral Content) levels were significantly higher in the OVX + Sema4D + leptin and OVX + Sema4D + MT groups compared to OVX + NL, while Tb.Sp (Trabecular separation) levels were significantly lower. In contrast to the OVX group, the bone trabeculae in the OVX + Sema4D + leptin and OVX + Sema4D + MT groups had a relatively complete structure and tended to be organized closely. The amount of bone trabeculae grew drastically, whereas the proportion of TRAP-positive osteoclasts declined dramatically. BMP-2 and leptin were also elevated, while BALP and TRAP-5b activity was reduced.

**Conclusion:**

Leptin or melatonin improved Sema4d's role in trabecular bone microstructure, bone production, and repairment of trabecular bone loss in osteoporosis rats.

## Introduction

A prevalent age-related illness is osteoporosis. Osteoporosis has recently emerged as a major global public health concern due to the sharp rise in patient numbers [1]. Statistics show that osteoporosis affects more than 200 million people worldwide [2]. In China, the frequency of osteoporosis among those over 50 years old is around 19.2%, and it can reach 32.0% among those over 65 [3]. In the USA, more than 50 million people over 50 have osteoporosis, and every year, almost 1.5 million people have an osteoporotic fragility fracture, which is predicted to rise by one-third by 2030 [4]. Specifically at trabecular areas like the spine, ribs, and hip cortex, osteoporosis is characterized by substantial abnormalities in the quality and quantity of bone structures, leading to decreased bone strength and fracture resistance. Fragility fractures, which are strongly related to mortality and disability [5, 6], are more likely to occur as a result of decreased bone strength and fracture resistance [7]. Osteoclasts that break down too much bone and osteoblasts that produce too little new bone are two factors in the etiology of osteoporosis. Increased fragility and fracture are brought on by a loss of bone density brought on by an imbalance between the processes that produce and resorb bone. In order to raise bone density and create a new foundation for the treatment of osteoporosis, it is crucial for patients with the disease to decrease the hormones that negatively regulate bone metabolism and increase the hormones that positively regulate it [8].

Sema4D, a protein involved in brain signaling, belongs to the semaphorin family. It has been established that semaphorins, also known as immunological semaphorins, and immune control have a close association. According to reports, Sema4D is involved in a wide range of signal transduction pathways and performs a wide range of biological functions, including the control of B cells, T cells, and other immune cells as well as the development of the nervous system, platelet function, epithelial cell function, and endothelial cell function [9]. Sema4D has been discovered to be expressed on osteoclasts in recent years, inhibiting bone production and possibly contributing to the onset of osteoporosis and osteosclerosis [10]. On chromosome 7q31.3, a 16kD protein called leptin has 167 amino acids. Leptin is finally detected in the blood with only 146 amino acid residues after going through a number of changes [11]. Leptin regulates energy by working on the central nervous system, primarily through the negative feedback mechanism to regulate hunger and activate receptors on target cells. Leptin is mostly generated from adipose tissue. Leptin is also thought to play a role in the regulation of bone production, which makes it an intermediary chemical in the regulation of bone metabolism [12]. The pineal gland secretes melatonin, a neuroendocrine hormone that is dispersed in a variety of tissues and organs including the gastrointestinal system, ovaries, lymphocytes, macrophages, retina, and skin at night under the control of the suprachiasmatic nucleus of the hypothalamus [13]. The paracrine or autocrine effects of melatonin, which are generated from the pineal gland's exterior, function as a local antioxidant and are overlaid on neuroendocrine hormone responses [14, 15]. Melatonin has been shown to reduce skeletal muscle weakness, prolong physical performance, and treat mood problems [15]. Melatonin may be particularly crucial for bone growth and development, according to a report by Reiter R et al. that the concentration of bone marrow was higher than that of blood [16, 17].

Using an OVX rat model, the current work intends to investigate the effects and processes of Sema4D, Sema4D paired with leptin, and Sema4D combined with melatonin on bone metabolism in an effort to identify possible molecular targets for osteoporosis therapeutic treatment.

## Materials and methods

### Experimental animals

SD (Sprague Dawley) rats, 32 females, 200–220 g Zhejiang Weilihua Experimental Animal Technology Co., Ltd., License No. SCXK (Zhejiang) 2019-0001), Rearing Environment: Temperature 20–26 °C, Humidity 40%- 70%. All research was done in compliance with international standards for the treatment and use of laboratory animals, and needless harm to the animals was avoided at all costs [18].

### Laboratory reagents and instruments

Medicine refrigerator (BYC-310, Shandong Boke Biology), refrigerated freezer (BD/BC-415DKEM, Midea), slicer (BQ-318D, Bernard), electric blast drying oven (HGZF-101-1, Shanghai Yuejin Medical Instruments Co., Ltd.), thermostatic incubator (DHP-9054, Shandong Boke Biology), electric thermostatic incubator (DHP-9054, Shandong Boke Biology Industry Co., Ltd.); Pressure cooker (YS20ED, Suber), induction cooker (HK-22, Hanke Electric Appliance Factory, Dongfeng Town, Zhongshan City), fume hood; Microscope (CX43, OLYMPUS), microtome (2235, Leica), multifunctional enzyme marker (SuPerMax 3100, flash spectrum); First antibody: BMP-2 (AF5163, Affinity); leptin(DF8583, Affinity); Sema4d (PAB430Ra01, cloud clone); Second antibody: horseradish enzyme-labeled goat anti-rabbit IgG (H + L) (ZB-2301, Zhongshan Jinqiao, 1/100); Tartrate-resistant acid phosphatase staining solution (G1492, Solarbio); Rat tartrate-resistant acid phosphatase 5b (TRACP-5b) ELISA reagent (MM-70644R1, enzyme immunoassay); Rat bone alkaline phosphatase (BALP) kit (MM-0619R1, enzyme immunoassay); Melatonin (S20287) was purchased from Shanghai Yuanye Biotechnology Co., Ltd., with a relative molecular weight of 232.28 and a purity of more than 99%.

### Creation of adenovirus vector

Construct the PCR primer for the target fragment based on the sequence of the target gene Sema4D (NM 001,170,563.2, CDS: 2588 bp) and Leptin (NM 001,003,679.3, CDS: 2773 bp); As the carrier for digestion, the suitable restriction enzyme was chosen, and the pure linear carrier was extracted from the agarose gel; Then, perform PCR on the target fragment and recover the agarose gel to acquire the right fragment size; Link the linearization vector to the target fragment utilizing homologous recombination or the T4 technique; Transformed responsive DH5a or stbl3, covered with bacterial fluid and cultured for 12 to 16 h; Choose the moving monoclonal colony for validation; Choose the most appropriate positive clone for colony verification prior to sequencing; The extraction of plasmids was carried out on cloned samples with the right sequence. Anhui General Biology Co., Ltd. is responsible for the adenovirus packaging of the vector after the sequencing verification. The specific information on the viral vector is as follows [18]:

### Target Sema4D gene sequence

ATGAAGATGTGTGCCCCCGTCAGGGGGCTGTTCTTGGCCCTGGTGGCTGTGTGGAGGACCGCGGTGGCATTCGCCCCTGTGCCTCGGATCACCTGGGAGCACGGAGAGGTAGGTCTGGTGAAGTTTCACGAGCCAGGCATCTTTAACTACTCTTCCTTGCTGATGAGTGAAGACAAAGACACTCTGTATGTGGGTGCCCGGGAAGCTGTCTTTGCAGTGAATGCGCTGGACATCTCTGAGAAGCAACATGAGGTATACTGGAAGGTCTCTGAAGACAAAAAATCCAAGTGCGCAGAGAAGGGGAAATCAAAGCAGACGGAGTGCCTTAACTACATCCGAGTGCTGCAACCGCTTAGCAGCACTTCCCTCTACGTGTGTGGGACCAATGCGTTCCAGCCCACCTGTGACCACCTGAACTTGACCTCTTTCAAGTTTCTGGGGAAAAGCGAAGATGGCAAAGGAAGATGCCCCTTCGACCCCGCCCATAGCTACACATCCGTCATGGTCGGGGGAGAGCTCTACTCTGGGACTTCATATAATTTCTTGGGCAGCGAACCCATCATCTCTCGAAACTCTTCCCACAGTCCCCTGAGGACAGAGTACGCCATCCCTTGGCTAAACGAGCCTAGCTTCGTCTTTGCTGACGTGATCCACAAGAGCCCAGATGGTACAGAGGCTGAGGATGACAAGGTCTACTTCTTCTTTACGGAGGTGTCCGTGGAGTACGAGTTCGTCTTCAAGTTGATGATCCCGCGAGTTGCCAGGGTGTGCAAGGGCGACCAGGGCGGCCTGCGGACTTTGCAAAAAAAGTGGACCTCCTTCCTAAAGGCCAGACTGATCTGCTCCAGGCCAGACAGTGGCCTGGTCTTCAACATTCTTCAAGATGTGTTTGTGCTGAGGGCCCCGGGCCTCAAGGAACCTGTGTTCTATGCGGTCTTCACCCCACAGCTGAACAACGTGGGTCTGTCAGCGGTCTGTGCCTACACGCTGTCCACGGTGGAGGCCGTCTTCTCCCGAGGAAAGTACATGCAGAGTGCCACAGTGGAGCAGTCTCACACCAAGTGGGTACGCTACAATGGCCCAGTGCCCACTCCCCGGCCTGGAGCGTGTATCGACAGTGAGGCCCGGGCAGCCAACTACACCAGCTCCTTGAATCTCCCAGACAAAACGCTGCAGTTTGTCAAAGACCACCCTTTGATGGACGACTCGGTGACGCCAATAGACAACAGGCCGAAACTGATCAAAAAAGATGTCAACTACACCCAGATAGTGGTAGACAGGACCCAGGCCCTGGATGGGACCTTCTACGACGTCATGTTCCTCAGCACAGACCGGGGCGCTCTGCATAAAGCTGTCATCCTTGCAAAAGAGGTACACGTGGTTGAGGAGACCCAACTCTTCCAGGACTTCGAACCGGTCCTGTCTCTGCTGCTATCATCAAAGAAGGGGAGGAAGTTTGTCTATGCTGGCTCCAACTCAGGAGTGGTCCAAGCTCCCCTGGCCTTCTGCGGAAAGCACAGTAGCTGTGAAGACTGTGTGCTAGCACGGGACCCCTACTGCGCCTGGAGCCCAGCCATCAAGGCCTGTGTTACCTTGCACCAGGCAGAGGGCTCTAGCAGGGGCTGGATTCAGGACATGAGTGGCGACACGTCCTCGTGCCTGGATAAGAGTAAAGAAAGTTTCCATCAGCATTTTTTCAAGCACGGCGGCACAGCAGAACTCAAGTGTTTCCAAAAGTCCAACCTGGCCCGGGTGGTGTGGAAGTTCCAGAACGGCGAGTTGAAGGCTGTGAGTCCCAAGTATGGCTTTGTGGGCAGGAAGCACCTGCTCATCTTTAACCTGTCAGACGGAGACAGCGGTGTGTACCAGTGCCTGTCAGAGGAAAGGGTCAGGAATAAAACGGTCTCCCAGCTGCTCGCCAAGCACATCCTGGAAGTGAAAATGGTAGCTCGGATCCCCCCATCACCTACCTCACAGACTGCTCAGACAGAAGGTAGTAGGATCACATCCAAAATGCCTGTGGCGTCTACCCAGGGGTCCTCTCCCCCTACCCCGGCTCTGTGGGCAACCTCCCCCAGGGCTGCCACCCTACCTCCCAAGTCCTCCTCCACCGGCACGTCCTGTGAACCAAAAATGGTCATCAACACGGTCCCACAGCTCCACTCGGAGAAGACAGTGTATCTCAAGTCCAGTGACAACCGCCTGCTCATGTCTCTCCTCCTCTTCCTCTTTGTCCTCTTCCTCTGCCTCTTTTCCTACAACTGCTACAAGGGCTACCTGCCCGGACAGTGCTTAAAGTTCCGCTCAGCCCTGCTGCTCGCAAAGAAAAAACCCAAGTCAGAGTTCTCTGACCTGGAGCAGAGTGTGAAGGAGACGCTGGTAGAACCTGGGAGCTTCTCGCAGCAGAACGGCGACCAGCCCAAGCCAGCCTTGGATACCGGCTATGAAACCGAGCAGGACACTATCACCAGCAAGGTCCCCACCGATCGAGAGGACTCGCAACGTATCGACGAGCTCTCCGCCAGGGACAAACCGTTTGATGTCAAGTGTGAACTCAAGTTTGCAGACTCGGATGCCGACGGGGACTGA.

### Target leptin gene sequence

ATGTGCTGGAGACCCCTGTGCCGGTTCCTGTGGCTTTGGTCCTATCTGTCCTATGTTCAAGCTGTGCCTATCCACAAAGTCCAGGATGACACCAAAACCCTCATCAAGACCATTGTCACCAGGATCAATGACATTTCACACACGCAGTCGGTATCCGCCAGGCAGAGGGTCACCGGTTTGGACTTCATTCCCGGGCTTCACCCCATTCTGAGTTTGTCCAAGATGGACCAGACCCTGGCAGTCTATCAACAGATCCTCACCAGCTTGCCTTCCCAAAACGTGCTGCAGATAGCTCATGACCTGGAGAACCTGCGAGACCTCCTCCATCTGCTGGCCTTCTCCAAGAGCTGCTCCCTGCCGCAGACCCGTGGCCTGCAGAAGCCAGAGAGCCTGGATGGCGTCCTGGAAGCCTCGCTCTACTCCACAGAGGTGGTGGCTCTGAGCAGGCTGCAGGGCTCTCTGCAGGACATTCTTCAACAGTTGGACCTTAGCCCTGAATGCTGA.

The following is the map of the Semad4 adenovirus overexpression vector:
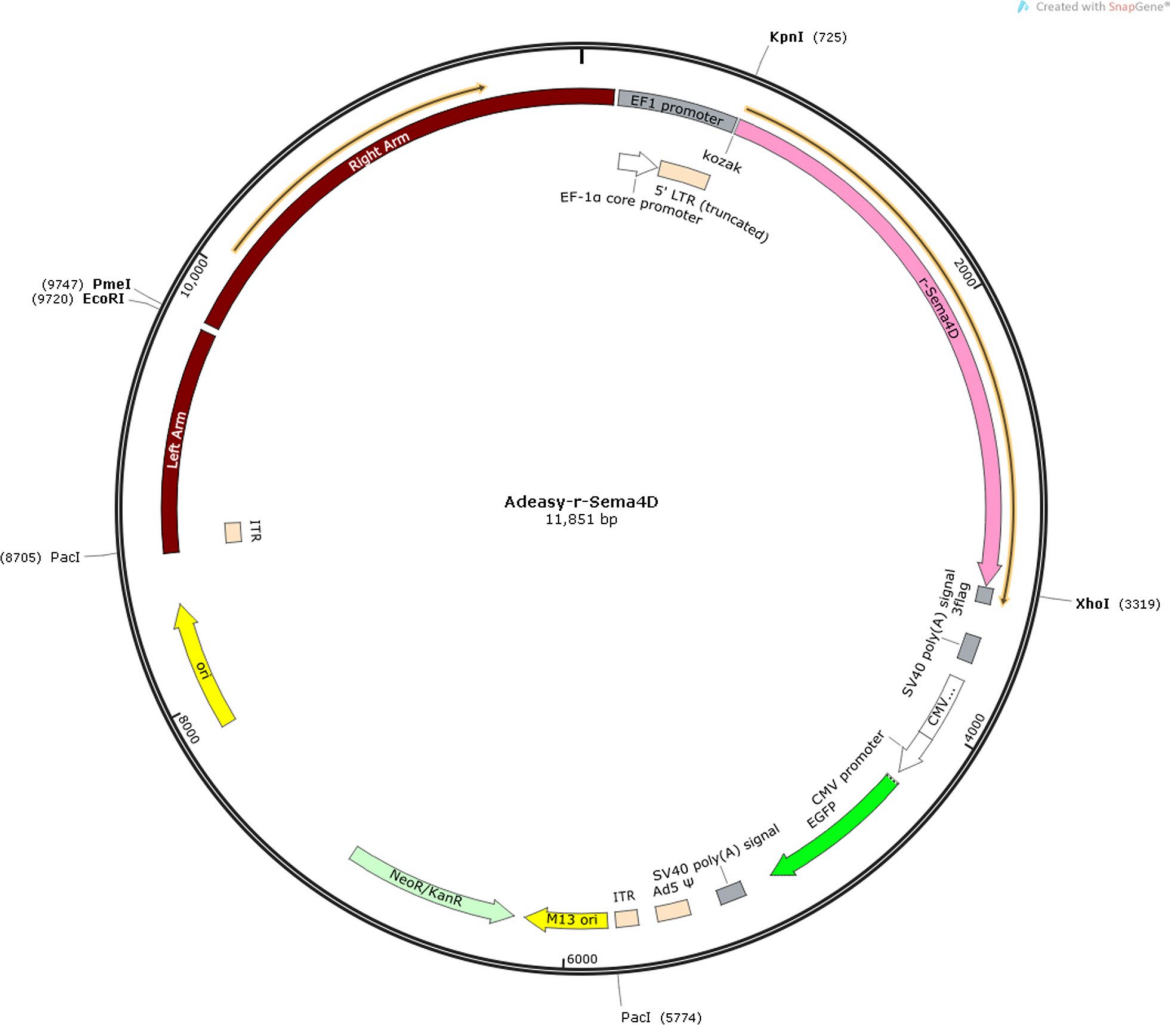


The vector map of Leptin adenovirus overexpression is as follows:
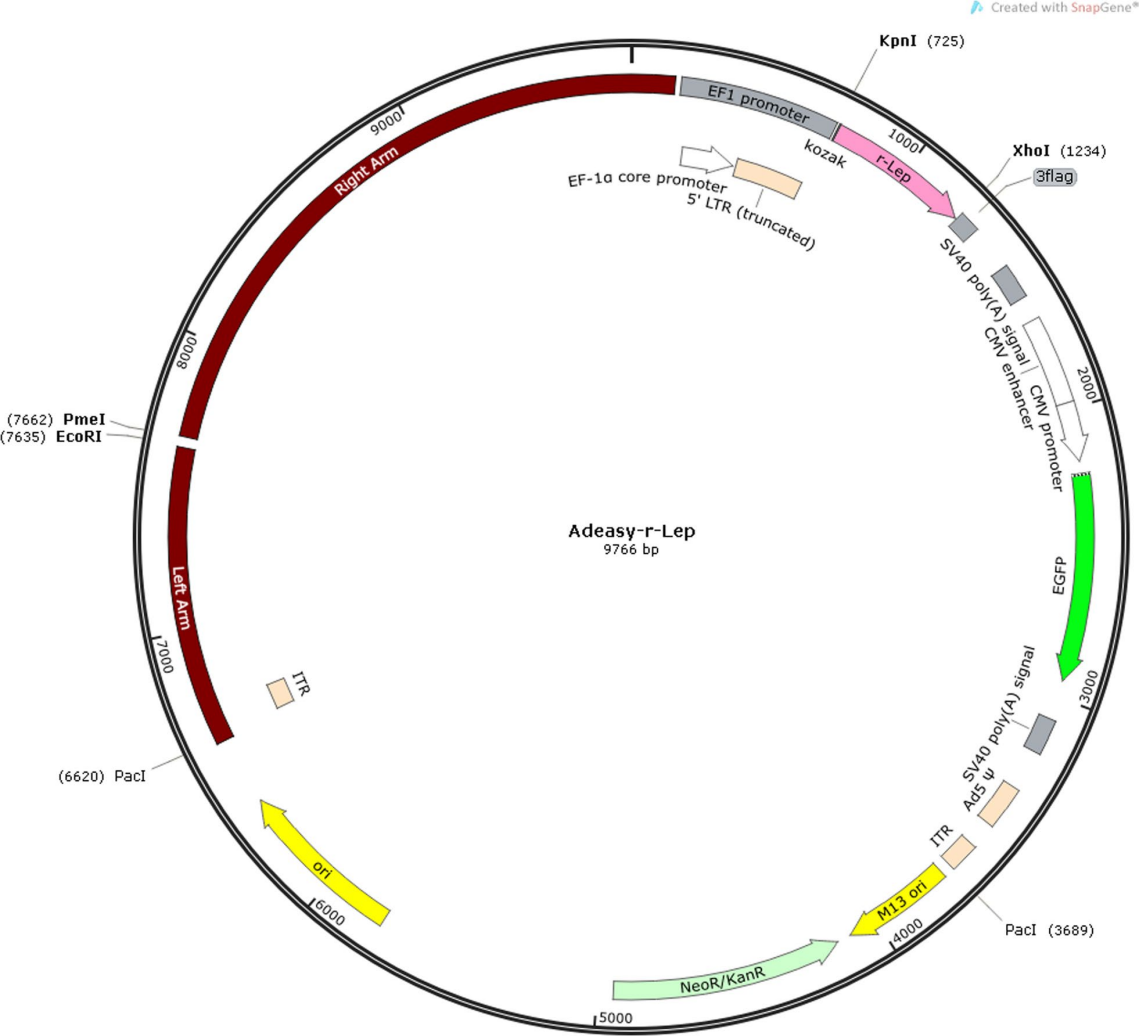


### OVX modeling and grouping

The occurrence and progression of osteoporosis are significantly influenced by the estrogen released by the ovaries. Rats' decreased estrogen secretion following ovariectomy results in a weaker suppression of osteoclasts. Bone production is outpaced by bone absorption. Because the bone is in a high stage of transition, the mass of the bone cannot be balanced, which causes more bone to be lost. The best model to research postmenopausal osteoporosis is the ovariectomized osteoporosis model [19, 20]; According to the technique of Li et al. [21], after anesthesia and under aseptic conditions, create a 1-2 cm incision in the lateral abdomen, and then passively separate it from the skin-fascia-muscle in succession using scissors. The white and moist fat mass is visible, and when the fat mass folds are removed, the pink and bright ovaries like cauliflower are revealed. Remove the uterus and both ovaries bilaterally. After cleansing the incision, the skin and underlying layer should be sutured in two layers. When the rat is awake, place it back in the clean cage and raise it in the feeding room. Observe and record the rats' health and deaths on a regular basis. Each rat was injected with 20,000 U/kg of penicillin three days after surgery to prevent infection. After bilateral oophorectomy, intragastric administration is used. It takes 1–3 months to induce bone loss in rats after OVX operation, and eventually leads to osteoporosis [22], and we carried out model validation on the 62nd day after modeling. Micro-CT and HE staining showed that the number of bone trabeculae in the model group was small and the density was low, indicating that the model was successfully constructed; therefore, group 6 was treated with melatonin by gavage on the 62nd day after modeling, once a day for 28 days, 50 mg/kg, once a day [23]. Adenovirus injection: four days prior to sampling, the rats were anesthetized by intraperitoneal injection, the lower limbs were shaved and disinfected, the open patella was pulled to one side along the direction of the knee joint, the tibial platform was exposed, a small hole was drilled on the tibial platform with a dental drill, 50UL adenovirus was extracted and injected into the bone marrow cavity, and the small hole was qPCR was used to detect the Sema4D or leptin gene expression in tibial tissue in order to confirm the efficacy of virus transfection. In accordance with the above treatment, they were divided into the following groups: (1) normal control group (sham operation) (Control), (2) OVX model group (OVX), (3) OVX model + Sema4D gene adenovirus over-expression empty group (OVX + NL), and (4) OVX model + Sema4D gene adenovirus over-expression vector group (OVX + Sema4D). (5) OVX model + Adenovirus over-expression vector for the Sema4D gene + Adenovirus over-expression vector for the leptin gene (OVX + Sema4D + leptin) (6) OVX model + Sema4D gene over-expression adenovirus vector + melatonin intervention group (OVX + Sema4D + MT).

### The 3D bone structure was detected using the high-resolution micro-CT system

Bai et al. [24] describe the micro-CT system's technique for detecting 3D bone structure. After killing rats, the soft tissue around the tibia was removed under sterile conditions, and the tibia was scanned and reconstructed by Micro-CT. Then the six groups of indicators were compared, including bone mineral density (BMD, g/cm3), bone mineral content (BMC, g), trabecular thickness (Tb.Th), trabecular number (Tb.N), trabecular spacing (Th.Sp), and bone volume/total volume (BV/TV, %).

### ELISA detection of BALP, TRAP-5b, and estrogen

Obtain serum samples and separate the supernatant for testing using a centrifuge. After treatment, BALP, TRAP-5b, and estradiol were identified according to the processes outlined in the instructions for the respective ELISA kit [25–27]. Use 0.05 M PH 9.0 carbonate-coated buffer to dilute the antibody to 1–10 g/ml protein concentration. Overnight at 4 °C, add 0.1 mL to each polystyrene board's reaction hole. The following day, remove the solution from the hole and wash it three times for three minutes with washing buffer. Add 0.1 mL of diluted test sample to the coated reaction hole and incubate for 1 h at 37 °C. wash. In each reaction well, add 0.1 mL of freshly diluted enzyme-labeled antibody. Wash after incubating at 37 °C for 0.5 to 1 h. Add 0.1 mL of temporarily produced TMB substrate solution to each reaction well and incubate at 37 °C for 10 to 30 min. To stop the reaction, add 0.05 mL of 2 M sulfuric acid to each reaction well. Using an enzyme marker, determine the OD of each hole. The concentration of each factor in the sample is determined by comparing the sample to the standard curve.

### HE staining

Following the collection of tibia tissues from each animal, the tissues were washed in water for two hours. After dehydration with various concentrations of ethanol solution, tissues were dehydrated with xylene until translucent, followed by embedding for one hour and slicing. The slides were then roasted, dewaxed, hydrated, bathed in distilled water, colored in a hematoxylin aqueous solution for three minutes, and then differentiated using a hydrochloric acid ethanol differentiation solution for fifteen seconds. After being cleaned briefly with water and blue-returning solution for 15 s, slides were rinsed with water and stained with eosin (G1100, Solarbio, China) for three minutes. The inverted microscope was then utilized to capture images (OLYMPUS, Japan).

### TRAP staining

Sections of paraffin were deparaffinized for 5 min, then progressively incubated in 100% ethanol for 5 min, 90% ethanol for 2 min, and 70% ethanol for 2 min. The TRAP staining solution was then added for fixation at 4 °C for 60 s. After being washed with water, slices were immersed in TRAP incubation solution at 37 °C for 45–60 min, then stained with methyl green for 2 to 3 min. Pictures were captured using a microscope (CX43, OLYMPUS, Japan).

### Immunohistochemical assay

The slides were washed in PBS for one hour before being incubated overnight with 10% goat serum. The slides were then incubated with primary antibodies against BMP-2 (1:100, AF5163, Affinity, USA) or leptin (1:100, DF8583, Affinity, USA) at 4 °C for 24 h, after which they were washed with PBS. The secondary antibody (1:100, ZB-2301, SolelyBio, China) was then added, followed by a 24-h incubation at 4 °C, rinsing, and DAB staining. Finally, photos were captured with an inverted microscope (CX43, OLYMPUS, Japan).

### Statistical analysis

For statistical analysis, SPSS 20.0 software was used. Each experiment was conducted three times. The mean standard deviation was used to express the quantitative outcomes (X $\pm$ S). The quantitative results of the two groups were compared using the T test for independent samples. Using one-way ANOVA, the quantitative values of the numerous groups were compared. The S–N-K technique was utilized to compare the two groups. Inspection level α = 0.05 and P < 0.05 indicate a statistically significant difference.

## Results

### The establishment of OVX model in rats and the identification of the transfection of adenovirus

**Fig. 1:**
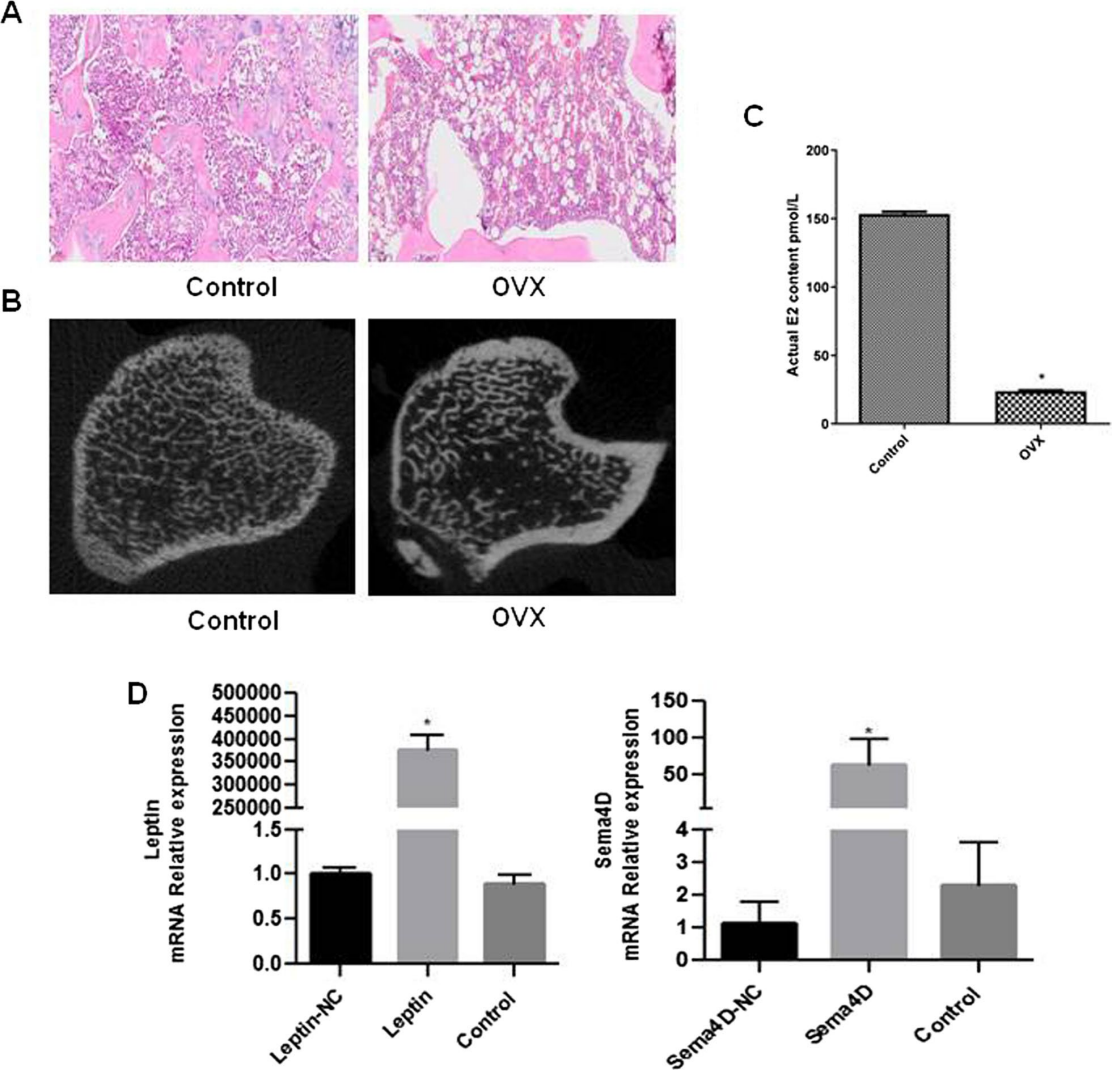
**A** HE staining for bone morphology observation; **B** Micro-CT scanning analysis of rat tibia samples; **C** The changes of estradiol in serum were detected by ELISA; **D** The expression of Sema4D and leptin genes in tibial tissue was detected by qPCR to verify the efficiency of virus transfection (*p < 0.05 vs. Control)

As shown in Fig. 1A, the tibia trabeculae in the control group were dense and interwoven. In the OVX group, the number of bone trabeculae was dramatically reduced, and the bone trabeculae were thinned and broken. Rat bilateral tibial specimens were scanned and analyzed using Micro-CT. As depicted in Fig. 1B, the number of bone trabecula was drastically reduced in the OVX group, concurrent with a decrease in bone density. In addition, OVX rats had significantly higher E2 levels compared to controls. These results indicated that the OVX paradigm was successfully implemented in rats. As shown in Fig. 1D, Sema4D and leptin were significantly upregulated in OVX rats compared to controls.

### The impact of Sema4d combined with leptin or melatonin on the microstructure of trabecular bone in tibia of OVX rats

**Fig. 2:**
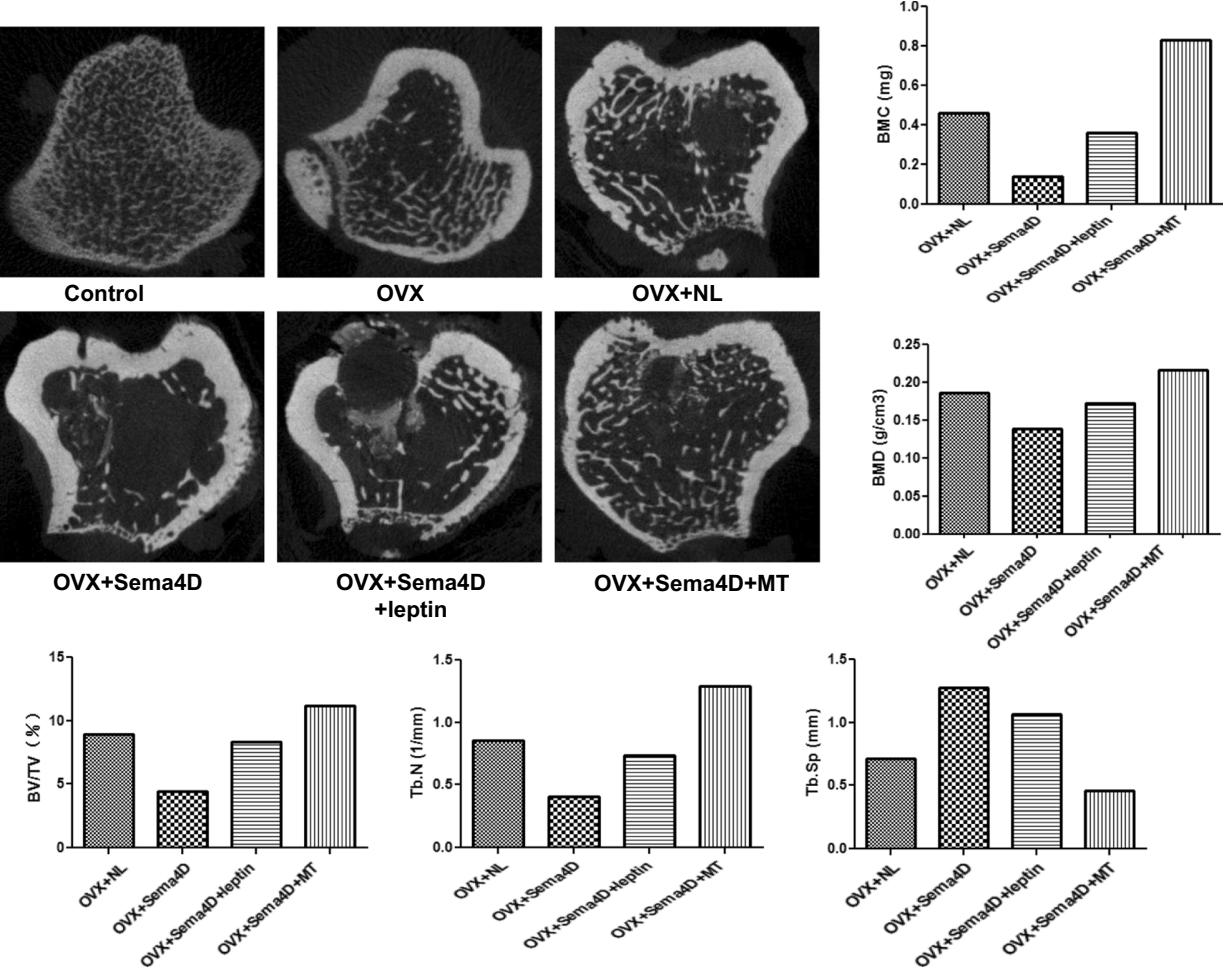
Depicts the effect of Sema4d gene overexpression in conjunction with leptin overexpression or melatonin intervention on the microstructure of osteoporotic rats' tibial trabecular bone

As demonstrated in Fig. 2, the levels of V/TV, Tb.N, BMD, and BMC were significantly elevated in the OVX + Sema4D + leptin and OVX + Sema4D + MT groups as compared to the OVX + NL group, but the level of Tb.Sp decreased dramatically.

### The impact of Sema4d combined with leptin or melatonin on the pathological changes in tibia tissues of OVX rats

**Fig. 3:**
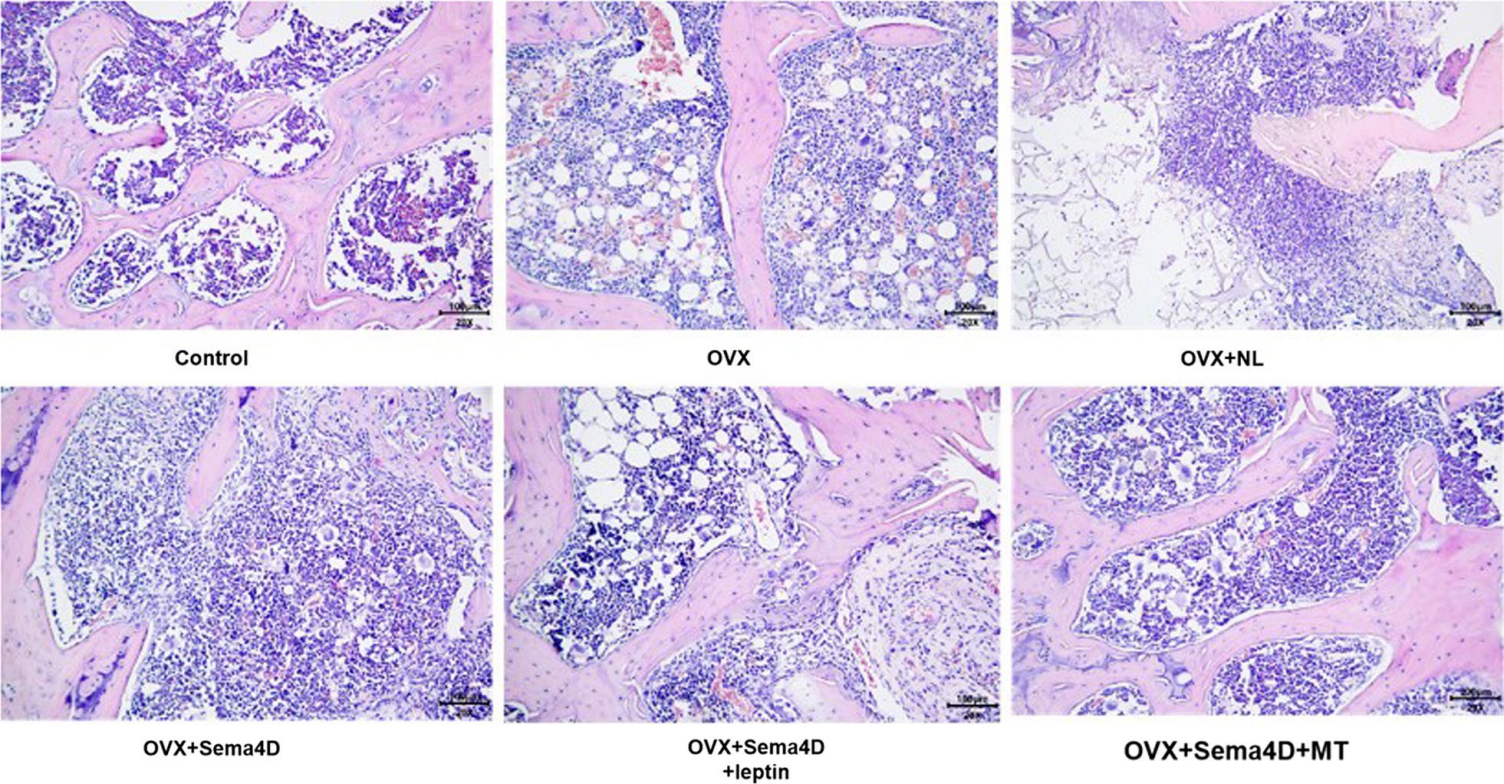
Observation of tibial bone shape in rats with osteoporosis following Sema4d gene overexpression in conjunction with leptin overexpression or melatonin intervention

As shown in Fig. 3, the structure of bone trabecula was stable and arranged orderly in the control group. Disrupted structure of randomly arranged trabecula and decreased number of bone trabecula were observed in the OVX, OVX + NL, and OVX + Sema4D groups. Compared to the OVX group, relatively tightly arranged integrated structure of bone trabecula and increased number of bone trabecula were observed in the OVX + Sema4D + leptin and OVX + Sema4D + MT groups.

### The impact of Sema4d combined with leptin or melatonin on the osteoclast differentiation in tibia tissues of OVX rats

**Fig. 4:**
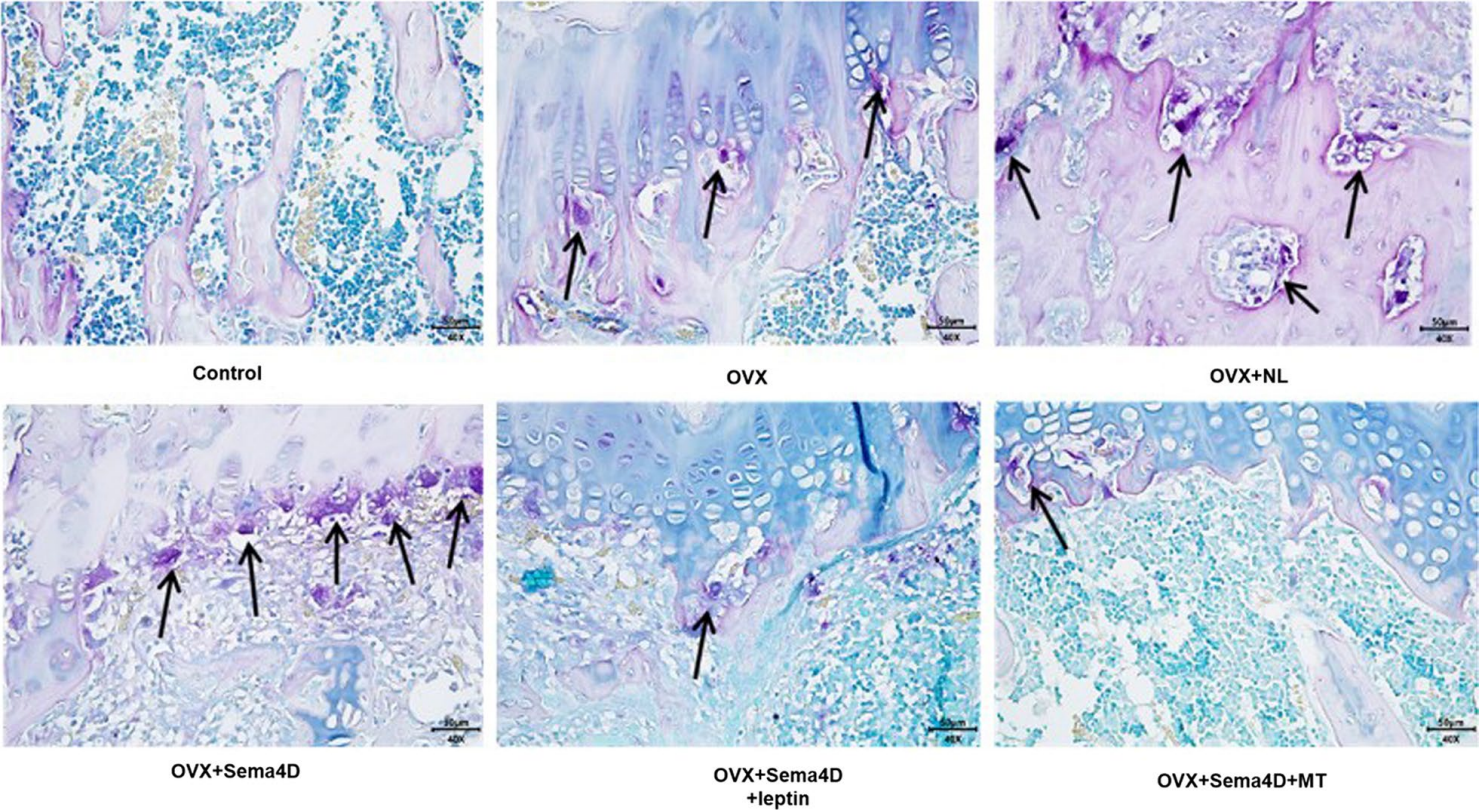
Depicts the effect of Sema4d gene overexpression in conjunction with leptin overexpression or melatonin intervention on the differentiation of osteoclasts in rats with osteoporosis

TRAP staining was used to evaluate the number of osteoclasts in metaphysis at the upper end of the tibia. Compared to control, the number of TRAP positive osteoclasts was dramatically increased in the OVX, OVX + NL, and OVX + Sema4D groups, among which the highest number of osteoclasts was observed in the OVX + Sema4D group. Furthermore, compared to the OVX group, dramatically declined TRAP positive osteoclasts were observed in the OVX + Sema4D + leptin and OVX + Sema4D + MT groups (Fig. 4).

### The impact of Sema4d combined with leptin or melatonin on the level of BMP-2 and leptin in tibia tissues of OVX rats

**Fig. 5:**
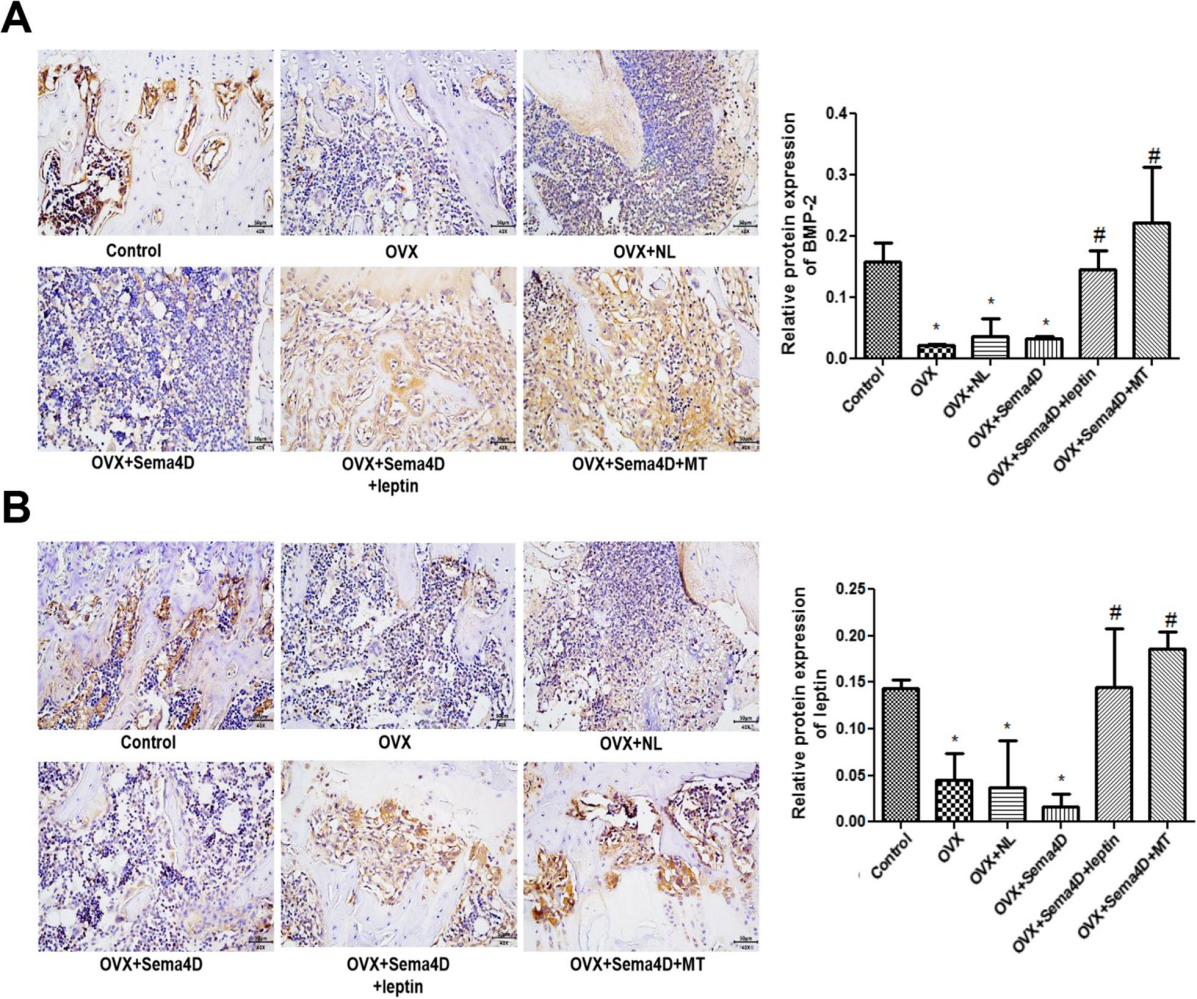
**A** Immunohistochemical detection and quantitative analysis of BMP-2 protein expression in bone tissue; **B** Immunohistochemical detection and quantitative analysis of leptin protein expression in bone tissue (* p < 0.05 vs. Control, # p < 0.05 vs. OVX)

As shown in Fig. 5, compared to control, extremely declined level of BMP-2 and leptin was observed in the OVX, OVX + NL, and OVX + Sema4D groups. Compared to the OVX group, dramatically elevated level of BMP-2 and leptin was observed in the OVX + Sema4D + leptin and OVX + Sema4D + MT groups.

### The impact of Sema4d combined with leptin or melatonin on the activity of BALP and TRAP-5b in OVX rats

**Fig. 6:**
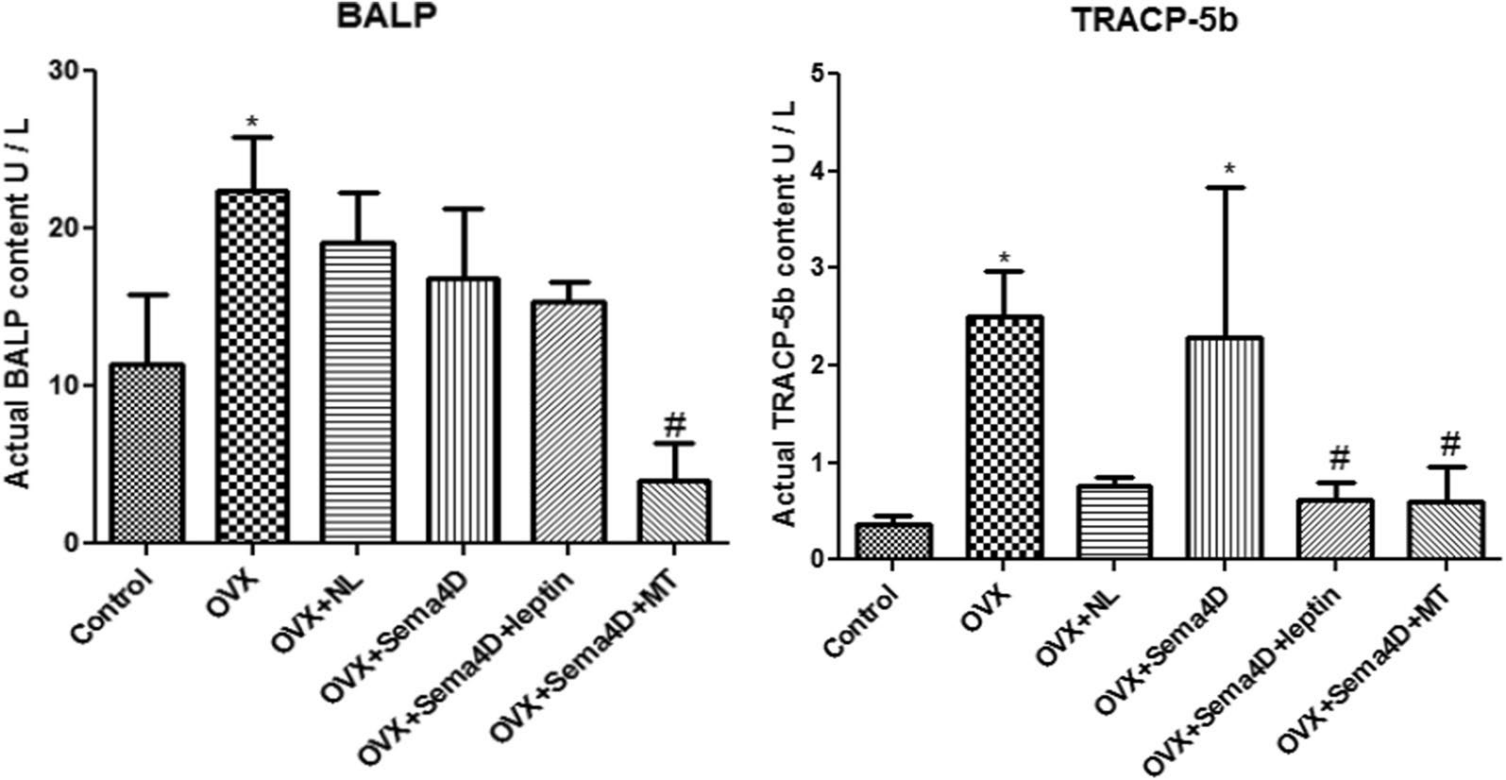
The effects of Sema4d gene overexpression in conjunction with leptin overexpression or melatonin intervention on BALP and TRAP-5b activities in rats with osteoporosis (* p < 0.05 vs Control, # p < 0.05 vs. OVX)

As shown in Fig. 6, compared to control, markedly elevated activity of BALP and TRAP-5b was observed in the OVX, OVX + NL, and OVX + Sema4D groups. Compared to the OVX group, dramatically reduced activity of BALP and TRAP-5b was observed in the OVX + Sema4D + leptin and OVX + Sema4D + MT groups.

## Discussion

Sema4D plays a crucial function in the regulation of osteoclast formation. Sema4D expression is increased during osteoclast proliferation [10]; Negishi-Koga et al. discovered that semaphorin 4D (Sema4D) expressed in osteoclasts is an axon directing protein that suppresses bone production. Sema4d silent animals displayed enhanced bone development and sclerosis compared to mice of the wild type [28]. Sema4D can increase osteoclast production and differentiation [29]. Together with our experimental findings, we found that overexpression of Sema4D can exacerbate osteoporosis damage; therefore, we conclude that Sema4D regulates the differentiation of osteoblasts in osteoporosis and is one of the major factors that cause osteoporosis.

In the present study, OVX was used to construct an osteoporosis rat model. Pathological results showed that the number of tibial trabeculae in OVX rats was significantly reduced, and the bone trabeculae were thin and fractured with low density, accompanied by an increased serum E2 level. These results were consistent with the research reports of Zhao [30] and Wang [31]. After the overexpression of Sema4D, the microstructure of tibial trabeculae was significantly damaged, the arrangement was irregular, the number of bone trabeculae was reduced, the number of osteoclasts was significantly increased, and the osteogenic-related indicators were significantly decreased. These results suggested that Sema4D might aggravate the degree of osteoporosis by increasing the number of osteoclasts, which was close to results reported by Takako [10].

Leptin is reported to promote the proliferation of osteoblasts and the transformation of bone marrow stromal cells into osteoblasts. In previous experiments, an extremely repressed long-bone growth, reduced bone surface area, and decreased mineral and bone density level were observed in leptin deficient mice which were dramatically rescued by the injection of leptin, indicating that leptin played an important role in the differentiation and maturation of osteoblasts [32]. It is reported that the increase in leptin level is positively correlated with the number of bone mineralized nodules in osteoblasts. Furthermore, the targets of leptin are found on osteoblasts, which further indicates that leptin exerts a positive effect on the metabolism of osteoblasts. Bone marrow mesenchymal stem cells (BMSCs) proliferate in large quantities in vivo and differentiate into a variety of cell types, such as osteoblasts and adipocytes. The differentiation of BMSCs to adipocytes and osteoblasts is reported to be facilitated by leptin [33], which further increases bone matrix, and affects the metabolism of bone cells. In the present study, after increasing the level of leptin in Sema4D-overexpressed OVX rats, significant improvement of tibia bone trabecular microstructure and arrangement of bone trabecular, increased number of bone trabecular, and elevated level of osteogenesis-related factors were observed, suggesting that leptin significantly reversed the pathological changes in tibia of Sema4D-overexpressed OVX rats. The functional mechanism of leptin might be associated with the receptor activator of nuclear factor-κB (RANK)/osteoprotegerin (OPG)/ receptor activator of NF-κB ligand (RANKL) axis. Studies have reported that OPG competitively binds RANK with RANKL to affect osteoclast activity, which is upregulated by leptin, thereby inhibiting the activation and maturation of osteoclasts, reducing bone resorption and bone destruction, and regulating bone metabolism [34, 35]. In the present study, the number of osteoclasts was significantly reduced after leptin overexpression in Sema4D-overexpressed OVX rats, which further verified that the improvement effect of leptin on osteoporosis might be related to the inhibition of osteoclast activation.

The effect of melatonin on primary osteoporosis has been widely reported. St Hilaire et found that melatonin levels were lower in premenopausal women and were accompanied by increased bone resorption [36]. Maria reported that melatonin activated melatonin receptor (MR) by inhibiting RANKL signaling to promote the generation of osteoblasts [37]. St Hilaire investigated the effect of melatonin on bone mineral density of postmenopausal women with osteoporosis in the past 10 years. It was found that melatonin increased BMD of femoral neck and lumbar spine in both perimenopausal and postmenopausal women. Combined treatment with melatonin improves scores at the femoral neck and lumbar spine, and melatonin may increase osteocalcin in both perimenopausal and postmenopausal women [38]. Due to its unique antioxidant, anti-inflammatory, and immunomodulatory functions, melatonin possesses great medical potential. It is an ideal method to treat osteoporosis by enhancing the activity of osteoblasts and inhibiting the formation of osteoclasts. A series of studies have reported the function of melatonin on bone metabolism. Zhu et al. found that melatonin effectively inhibited the osteolysis around the prosthesis caused by titanium particles [39]. Zhang et al. claimed that melatonin restored the osteogenic differentiation ability of stem cells damaged by titanium particles [40]. Chen et al. found that melatonin improved the osteogenic differentiation potential of BMSCs derived from osteoporotic rats [41]. In the present study, melatonin was used for the treatment of Sema4D overexpressed OVX rats. Results showed that in Sema4D overexpressed OVX rats, the structure of bone trabeculae was relatively complete, the arrangement of the bone trabeculae tended to be neat, the number of bone trabeculae was increased, the number of osteoclasts was decreased significantly, and the secretion level of BMP-2 and other osteogenic indicators was elevated significantly by the administration of melatonin. These results indicated that melatonin significantly reversed the aggravation of lesion caused by Sema4D overexpression in OVX rats.

In conclusion, leptin and melatonin reversed the activation of osteoclasts caused by overexpression of Sema4D, which intensified the pathological process of osteoporosis. The target molecules for the clinical therapeutic development of osteoporosis were offered by the current study as potential and trustworthy candidates.

## Data Availability

The experimental data will be available on the request.
